# The impact of bilateral facetectomy on the instantaneous helical axis of the functional thoracic spinal unit T4-5 during axial rotation

**DOI:** 10.1080/23335432.2021.1958059

**Published:** 2021-08-05

**Authors:** Paul Jonathan Roch, Dominik Saul, Nikolai Wüstefeld, Stefan Spiering, Wolfgang Lehmann, Lukas Weiser, Martin Michael Wachowski

**Affiliations:** a Department of Trauma Surgery, Orthopaedics and Plastic Surgery, University of Göttingen, Göttingen, Germany; b Kogod Center on Aging and Division of Endocrinology, Mayo Clinic, Rochester, MN, USA; c Praxis Für Zahnheilkunde, Alexander Thiemann Und Nikolai Wüstefeld (Ang. ZA), Bad Driburg, North Rhine-Westphalia, Germany; d DUO – Duderstadt Trauma Surgery and Orthopaedics, Duderstadt, Lower Saxony, Germany

**Keywords:** Thoracic spine, axial rotation, kinematics, facetectomy, instantaneous helical axis

## Abstract

The location of the instantaneous helical axis (IHA) and the impact of the facet joints (FJ) on the kinematics in the thoracic spine remain inconclusive. This study aimed to examine the IHA in the functional spinal unit (FSU) T4-5 during axial rotation in intact conditions and after bilateral facetectomy. Four human T4-5 FSUs were examined with an established 6D measuring apparatus in intact conditions and after bilateral facetectomy. The IHA’s parameters migration, location, and direction in the horizontal plane were calculated. Defined preloads in different positions were applied. Under the intact conditions, the IHA migrated about 4 mm and from one to the contralateral side according to the applied preload. The location of the IHA was observed in the anterior part of the spinal canal. After bilateral facetectomy, the location of the IHA shifted ventrally about 10 mm compared to the intact conditions. Under intact conditions, the direction of the IHA was minimally dorsally reclined. After bilateral facetectomy, the IHA was significantly more ventrally inclined. The study determined the location of the IHA under intact conditions at the anterior part of the spinal canal. The IHA of the FSU T4-5 is substantially influenced by the guidance of the FJs.

## 1 Introduction

In thoracic spine surgery, consideration of biomechanical characteristics is indispensable in terms of its impact on the surgical outcome and the risk of complications after surgery (Nicholls et al. 2017; Oe et al. 2019). The locations of the rotational axes in the thoracic spine under axial rotation have been observed to be at the posterior end of the spinous process and at the anterior border of the vertebral body, and all the areas in between (Molnar et al. 2006). While one previous perspective assumed the locations of the instantaneous helical axis (IHA) of rotation at the anterior part of the spinal canal (Liebsch and Wilke 2018), a recently published study found the IHA locations to be slightly more anterior at the posterior part of the vertebral body (Liebsch et al. 2020).

Facetectomy is a procedure that can be performed in cases of facet joint (FJ) hypertrophy and osteophytes, if conservative treatment fails (O’Leary et al. 2018). Together with the ligamentum flavum and the anterior and posterior ligaments, FJs and their capsules account for the stability of the (thoracic) spine (Liebsch and Wilke 2018). Thus, facetectomy is regularly combined with stabilization since the procedure seems to be associated with spinal instability (O’Leary et al. 2018). While the impact of bilateral facetectomy on the rotational axes has been partially examined for the cervical and lumbar spine (Cusick et al. 1988; Zander et al. 2003), the influence of the FJs on the IHA of the thoracic spine remains unclear. Since the extension of a surgery in terms of a stabilization requires the insertion of screws and prolongs the operation time, thus increasing the risk for surgical complications (Gautschi et al. 2011; Schoenfeld et al. 2011), it is important that it be well justified based on biomechanical and clinical observations.

Given this background, the present study aimed to examine the IHA locations in intact conditions and to analyze the impact of bilateral facetectomy on IHA kinematics. Therefore, the study used a well-established 6D measuring apparatus to examine the kinematics during axial rotation in the functional spinal unit (FSU) T4-5 before and after bilateral facetectomy (Mansour et al. 2004; Wachowski et al. 2017; Roch et al. 2020a, 2020b). The reasons for analyzing the FSU kinematics with the 6D apparatus are its high spatial-temporal resolution and its motion-analyzing outcome parameter IHA. In contrast to parameters such as range of motion or stiffness, the IHA and its migration enable the comprehension of the complex motion properties of an FSU. Although the IHA has been analyzed by different approaches, including sequential radiography, optical systems, and finite element studies (Rousseau et al. 2006, 2008; Dugailly et al. 2010; Park et al. 2011; Crawford et al. 2012; Anderst et al. 2013, 2016), the 6D apparatus used in this study offers a comparably very high spatial-temporal resolution. The authors hypothesized that: (1) the location of the IHA is placed somewhere near the posterior part or even at the border of the vertebral body in the horizontal plane and (2) the resection of both FJs leads to an instability of the FSUs and a ventral shift in the locations of the IHA.

## 2 Methods

### Functional spinal units

**Figure 1.**
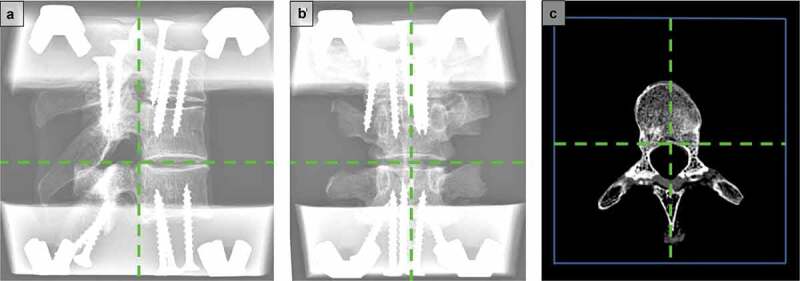
Imbedding of the Specimens. The figure shows the X-rays of an imbedded specimen in sagittal (**A**) and coronal (**B**) views and a horizontal view of the computer tomography scan (**C**). The green lines mark the virtual planes for the alignment of the vertebrae. They allowed centering of the vertebrae with the sagittal, coronal, and horizontal planes, respectively. A line running through two points in the middle of the spinous process in the horizontal plane defined the sagittal orientation, while the posterior border of the vertebral body determined the coronal orientation. The horizontal orientation was defined by the endplates. The blue framing in the horizontal view mark the frame of the bracket

Four T3-5 FSUs were used for the study (69.0 [SD 19.1] years, 2 females, 2 males). Interfering pathologies, such as osteochondrosis, spondylarthrosis, or other injuries, were excluded via computed tomography scans. The FSUs were imbedded in a formalin-containing solution. The limitations of formalin are discussed in the limitation section (Wilke et al. 1996; Holewijn et al. 2017). Soft and muscular tissues, ribs (through the costotransverse joint), vessels, and neural structures were removed, while vertebrae, ligaments, and capsules were retained. The FSUs were anchored to their fixtures with screws, then imbedded in brackets. To ensure sufficient freedom of movement, the T3 and T4 vertebrae were fixed to each other with screws, and T3 was embedded in the bracket serving as an elongation. After imbedding and fixation, computed tomography scans of each of the FSUs were repeated for later analyses. The positioning of the vertebrae in the brackets is crucial for the experiment. The vertebrae were centered in the brackets. Therefore, the sagittal orientation was defined by a line running through two points in the middle of the spinous process in the horizontal plane. The coronal position was determined by the posterior border of the vertebral body. The transition between the pedicle and posterior border of the vertebral body (the anterior end of the intervertebral foramen) of T4 was positioned in the coronal center of the brackets. The endplates of the segment of interest were aligned to the horizontal plane in the middle of the intervertebral disc (Figure 1).

### Experimental setup

**Figure 2.**
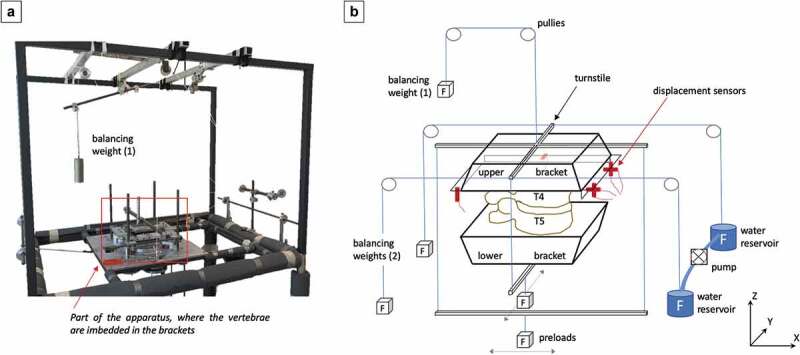
Experimental Setup. **A** Photograph of the 6D measuring apparatus. **B** The plan of the 6D measuring apparatus is depicted. The superjacent vertebrae (T3 and T4) are embedded in the mobile upper bracket, and the subjacent vertebra (T5) is embedded in the fixed lower bracket. The balancing weights are used to neutralize the weights of the upper bracket and the turnstile (1) and of the water pumps (2). The turnstile allows the application of the force system (F_z_, T_z_(t)). Preloads (200 N) were placed at five different positions: centric, dorsal, ventral, right, and left (each non-centric preload was 30 mm from the center). Six inductive displacement sensors (red) were mounted to the lower bracket

A well-established 6D measuring apparatus was used for the experiments (Mansour et al. 2004; Wachowski et al. 2013, 2017; Roch et al. 2020a, 2020b). A water pump system was used to apply an axial rotation with a cyclically varying torque-time function Tz(t) (frequency ≈$\frac{1}{120}Hz$). The torque was applied on the mobile superjacent vertebra, T3/4, while the subjacent vertebra, T5, remained fixed as a reference. When the system was at rest (T_x_ = 0), the forces were balanced. When water (6000 mL) was pumped from reservoir A to reservoir B and vice versa, the apparatus performed an axial rotation. Physiological interplay was simulated by defined preloads on the Y-/X-axis. The weight of the head and the upper thorax were simulated by preloads, which varied in position. There were five preload positions: One preload was positioned at the center and four preload positions in each direction, 30 mm from the center (dorsal, ventral, right, and left). Each preload was defined at 200 N (Figure 2).

Six inductive displacement sensors (Millimar 1310 inductive probe, Mahr GmbH, Göttingen, Germany [Mansour et al. 2004]) were mounted to the lower bracket and their tips touched the upper bracket with three orthogonally orientated glass plates. They were arranged in three orthogonal planes in a 3-2-1 configuration to record the spatial movements of the mobile superjacent vertebra (Figure 2B). The displacement of the sensors by impression and release of the tips allows the registration of the upper bracket’s spatial positions (T4). Equal amplitudes of the six displacement sensors under an applied torque signified the stable and constant movement necessary for data collection. The apparatus captures at least 400 positions in one cycle of Tz(t) with a spatial resolution of < 2.4 µm for axial rotation and < 10^−3^ degrees for rotation. Data from the displacement sensors was used to calculate the IHA (position, direction) and its migration rate as functions of the rotational angle α (Mansour et al. 2004).

For each FSU the center of resistance was determined as the point of the least sensor displacement during axial rotation. The axes of the device were defined as follows: The Z-axis ran vertically through the center of resistance. The X-axis was defined as normal to the sagittal, and Y-axis as normal to the coronal plane, both running through the Z-axis. The experiments began with the intact segments for all preload conditions, resulting in five runs (sequence: central, dorsal, ventral, right, left). Thereafter, bilateral facetectomy of T4-5 was performed by an experienced spine surgeon. The operative procedure was performed while the specimen remained in the apparatus, though the calibration was not affected. Then, the experiment was repeated for all five preload conditions, again resulting in five runs (sequence: central, dorsal, ventral, right, left).

### Data processing

The algorithm that was used to calculate the IHA is a modified version of the direct approach used to determine the IHA, and it was recently critically discussed (Kinzel et al. 1972; Roch et al. 2020a, 2020b). As in previous studies, the data refers to a small range of axial rotations to reflect physiological motions $\left(-1.0^{\circ }\leq \alpha \leq +1.0^{\circ }\right)$ (Wachowski et al. 2017; Roch et al. 2020a, 2020b). For the analyses, the mean values from the two rotational directions (right, left) were used. Two parameters were calculated: 1) The IHA direction, which is the inclination of the IHA in each dimensional plane and 2) the IHA migration in the axial plane. This migration was visualized as a path in the axial plane at Z = 0 cm. Mean IHA locations were defined as the IHA positions at α = 0° of axial rotation and were used for calculations (Wachowski et al. 2017; Roch et al. 2020a, 2020b). Spatial relationships were analyzed based on the IHA positions (Mansour et al. 2004). CT scans allowed for visualization of the IHA migration path.

### Coordinate system

Calculation of the IHA is based on the displacement of one coordinate system in relation to another (Roch et al. 2020a, 2020b). The center of the initial coordinate system was set in the right caudal posterior corner of the lower bracket (reference) and was gauged by the 6 displacement sensors. For further calculations, the center was re-adjusted to the center of the coordinate system as defined by the positioning of the FSUs as described above (X-/Y-/Z-axes): The axial plane was set through the middle of the intervertebral disc, the coronal plane was set at the posterior border of the vertebral body, and the sagittal plane through the X- and Z-axis. For the analyses and visualizations, the CT scans were adjusted using standardized brackets that enclosed the vertebrae; thus, they could be used as a reference. The points at which the IHA intersected with the axial plane in the vertical middle of the intervertebral disc determined the IHA positions.

### Statistics

Differences between IHA direction, position, and migration for each preload positions were calculated. The Wilcoxon test and Friedman test for paired observations were used due to the presence of nonparametric data. Post hoc tests and corrections for multiple testing were performed with the Bonferroni approach. SPSS Statistics software version 26.0 (IBM SPSS Inc., Chicago, IL, USA) and GraphPad Prism 8.2.1 (GraphPad Software, San Diego, CA, USA) were used. Values of *p* < 0.05 were considered to be statistically significant. The results in the tables are shown as median and quartiles (nonparametric data). For a better readability, data in the text is depicted as mean and SD. Statistical analyses were supervised by the Department of Medical Statistics, University of Göttingen.

The study was approved by the ethics committee of the University of Göttingen (No. 17/12/09).

## 3 Results

### Instantaneous helical axis position

**Figure 3.**
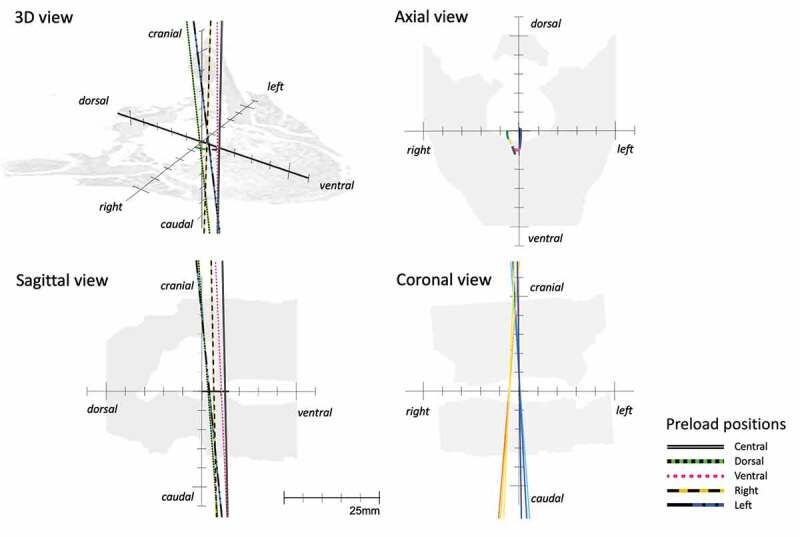
IHA under Intact Conditions. The IHA direction and migration under intact conditions are displayed for axial rotations of −1°, 0°, and +1° and IHA direction. Colors and styles of lines are used to define different preloads. In the coronal view, the IHA directionis depicted only under lateral preloading (right and left) using colors with different tones (−1° with a light color, 0° with a medium color, and +1° with a dark color). The horizontal plane shows the average IHA migration paths (different colors without specific line styles are used to define the preloads)

Under intact conditions, the location of the IHA (IHA positions at α = 0° of axial rotation) and under a central preload condition was at X = 6.1 (SD 7.3) mm and Y = −1.1 (SD 2.2) mm in the axial plane. Dorsal and left preload positioning led to an IHA location that was located significantly more dorsally compared to the central preload position (*p* = 0.010, Friedman test). Furthermore, the IHA location was significantly more rightwards under the dorsal preloading condition than under a ventral or right preloading condition (*p* = 0.010, Friedman test) (Table 1, Figure 3).

**Table 1.** IHA direction, migration path length, and positions under the intact conditions

**IHA direction (deg) [median (q1, q3)]**												
	**Preload** **Rotation**	**central**		**dorsal**		**ventral**		**right**		**left**		***p***
**Sagittal plane**	−1° (left)	−2.3 (−3.8, 2.4)		−2.9 (−10.1, 1.6)		−1.9 (−5.2, 1.8)	**l**	5.0 (1.1, 5.7)	**l**	−9.2 (−14.1, −1.8)	**vr**	**<0.001**
	0°	−0.7 (−3.5, 0.7)		−2.0 (−10, 1.4)		−2.5 (−5.3, 0.3)		0.0 (−3.9, 1.6)		−4.7 (−10.5, −0.6)		0.052
	+1° (right)	−0.6 (−3.9, 0.9)		−2.8 (−5.4, 2.6)		−3.2 (−5.5, −0.3)		−6.4 (−7.9, −2)		−1.1 (−5.1, 2.1)		0.153
**Coronal plane**	−1° (left)	−0.4 (−1.9, −0.1)		−6.2 (−9.6, −2.8)	**vr**	2.3 (0.8, 4.5)	**d**	2.4 (0.8, 3.3)	**d**	−5.6 (−7.4, −1.0)		**0.001**
	0°	1.5 (−1.3, 1.7)		−2.9 (−4.8, −0.4)	**vr**	2.0 (1.4, 2.7)	**d**	3.2 (2.5, 4.8)	**dl**	−4.8 (−5.6, −0.5)	**r**	**0.002**
	+1° (right)	3.4 (0.9, 4.2)		1.9 (1.4, 2.4)		0.6 (−0.4, 2.6)		4.1 (2.4, 6.5)	**l**	−1.8 (−3.2, 1.4)	**r**	**0.018**
**IHA migration path**												
	Length (cm)	3.6 (2.0, 7.5)		3.5 (1.6, 5.6)		3.2 (2, 4.7)		2.7 (2.1, 4.5)		4.8 (2.5, 8.7)		0.260
**IHA positions (0°)**												
	X (cm)	5.4 (−0.7, 13.1)	**dl**	1.2 (−2.9, 6.3)	**c**	4.8 (−0.9, 11.7)		3.1 (−5.5, 10.2)		1.9 (−1.7, 5.2)	**c**	**0.010**
	Y (cm)	−0.7 (−3.2, 0.8)		−3.0 (−5.1, −1.1)	**vr**	−0.4 (−3.4, 2.4)	**d**	−1.5 (−6.4, 0.9)	**d**	0.5 (−1.5, 2.0)		**0.010**
**c/d/v/r/l**: p < 0.05 versus central/dorsal/ventral/right/left												
IHA direction: Sagittal plane: > 0 indicates ventral inclination, < 0 indicates dorsal reclination Coronal plane: > 0 indicates inclination to the left, < 0 indicates inclination to the right IHA positions: X: > 0 indicates ventral position, < 0 indicates dorsal position referring to the coordinates system’s origin Y: > 0 indicates left position, < 0 indicates right position referring to the coordinates system’s origin												
Due to the presence of nonparametric data, median and quartiles (q1, q3) are shown: q1: the first quartile, which separates the lowest 25% of the data from the highest 75%. q3: the third quartile, which separates the highest 25% of the data from the lowest 75%.												

The IHA direction, migration path length, and positions for the intact conditions are displayed for the different preloads. Significant differences between the preload positions are marked with the corresponding letters: c: central, d: dorsal, v: ventral, r: right, l: left. Deviations in the IHA directions in the sagittal and coronal planes were mostly observed for the ventral and lateral preloads (left, right) in comparison to the dorsal preloads. While the IHA migration path length did not differ between the preload positions, the IHA positions were different between the dorsal and lateral preloads.

**Figure 4.**
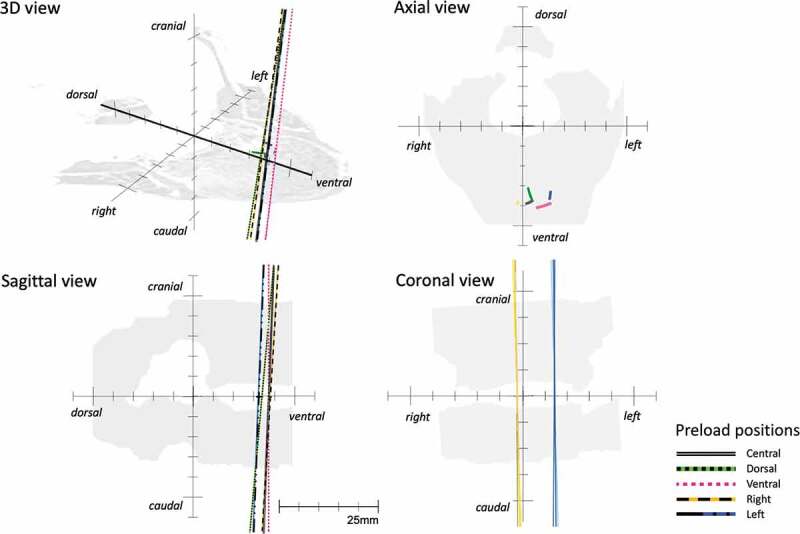
IHA after Bilateral Facetectomy. The IHA direction and migration after bilateral facetectomy are depicted for axial rotations of −1°, 0°, and +1° and IHA direction. Colors and styles of lines are used to define different preloads. In the coronal view, the IHA direction is depicted only under lateral preloading (right and left) using different color tones (−1° with a light color, 0° with a medium color, and +1° with a dark color). The horizontal plane shows the average IHA migration paths (different colors without specific line styles are used to define the preloads)

**Figure 5.**
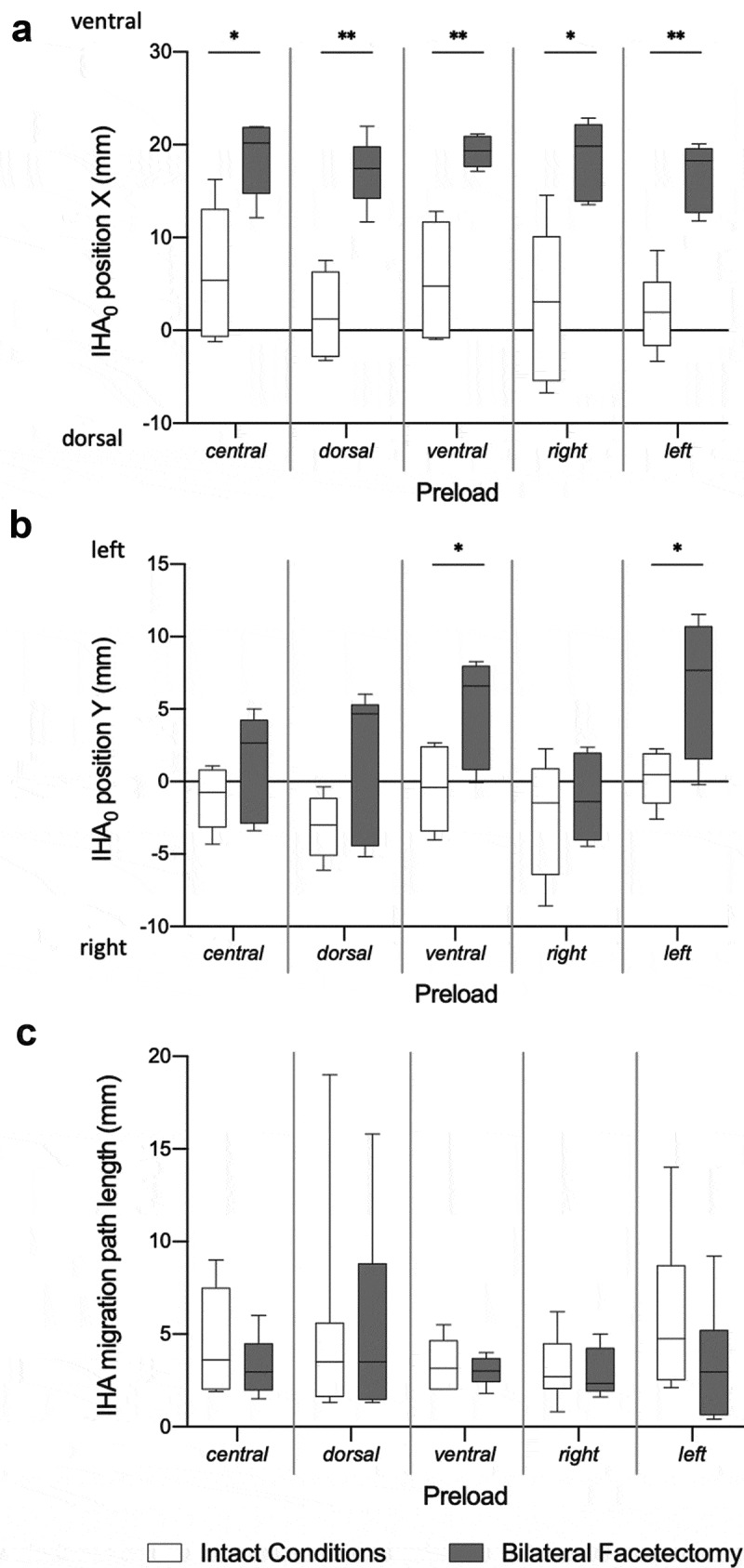
Differences in the IHA Migration Path Length and Positions. The IHA positions at 0° of axial rotation (A, B) and the IHA migration path length (C) under intact conditions and after bilateral facetectomy are depicted. The IHA_0_ position X shifted ventrally after bilateral facetectomy. Wilcoxon test, Dunn-Bonferroni correction for multiple testing, *: p < 0.05, **: p < 0.001

After bilateral facetectomy, the location of the IHA was at X = 18.7 (SD 4.0) mm and Y = 1.4 (SD 3.6) mm under the central preloading condition (Table 2, Figure 4). In comparison to the intact conditions, the IHA location after bilateral facetectomy shifted significantly more ventrally under all the preload positions and significantly more leftwards for the ventral and left preload positions (Figure 5A, B).

**Table 2.** IHA direction, migration path length, and positions after bilateral facetectomy

**IHA direction (deg) [median (q1, q3)]**											
	**Preload** **Rotation**	**central**	**dorsal**		**ventral**		**right**		**left**		***p***
**Sagittal plane**	−1° (left)	6.1 (4.9, 6.3)	11.3 (10.3, 18.3)	**v**	0.2 (0.1, 1.9)	**dl**	10.5 (10.1, 10.9)		5.3 (1.7, 6.1)	**v**	**0.006**
	0°	4.9 (4.1, 8.7)	9.4 (9.3, 16.4)	**v**	0.2 (0.1, 1.3)	**dr**	8.0 (7.5, 8.5)	**v**	5.9 (4.1, 6.1)		**<0.001**
	+1° (right)	4.2 (2.6, 10.2)	9.4 (8.5, 14.8)		0.2 (0.2, 1.2)		5.8 (5.2, 6.3)		6.7 (6.3, 7.1)		0.163
**Coronal plane**	−1° (left)	−2.9 (−7.9, −1.6)	−4.8 (−11.8, −3.8)	**v**	3.6 (2, 10.5)	**dl**	−3.3 (−9.3, −0.4)		−3.9 (−5.1, −3.1)	**v**	**0.004**
	0°	−2.0 (−4.0, −0.3)	−3.5 (−11.5, 0.2)	**vl**	2.5 (1.7, 7.9)	**dr**	−1.9 (−7.0, 0.6)	**v**	−1.0 (−4.1, −0.2)	**d**	**<0.001**
	+1° (right)	−0.1 (−0.5, 0.8)	−1.2 (−2.9, 2.9)		1.4 (1.2, 3.2)	**l**	−1.0 (−4, 1.6)		2.0 (−3.1, 2.2)	**v**	**0.021**
**IHA migration path**											
	Length (cm)	3.0 (2.1, 4.0)	3.5 (1.5, 6.5)		30.0 (2.6, 3.6)		2.4 (2.0, 4.0)		3.0 (0.7, 3.9)		0.920
**IHA positions (0°)**											
	X (cm)	20.2 (15.6, 21.9)	17.4 (15.0, 19.1)		19.4 (17.8, 20.9)		19.9 (14.0, 22.0)		18.3 (13.0, 19.4)		**0.040**
	Y (cm)	2.7 (−2.7, 4)	4.7 (−4.2, 5.1)		6.6 (1.1, 7.9)		−1.4 (−3.9, 1.9)		7.7 (2.1, 10.4)		**<0.001**
**c/d/v/r/l**: p < 0.05 versus central/dorsal/ventral/right/left											
IHA direction: Sagittal plane: > 0 indicates ventral inclination, < 0 indicates dorsal reclination Coronal plane: > 0 indicates inclination to the left, < 0 indicates inclination to the right IHA positions: X: > 0 indicates ventral position, < 0 indicates dorsal position referring to the coordinates system’s origin Y: > 0 indicates left position, < 0 indicates right position referring to the coordinates system’s origin											
Due to the presence of nonparametric data, median and quartiles (q1, q3) are shown: q1: the first quartile, which separates the lowest 25% of the data from the highest 75%. q3: the third quartile, which separates the highest 25% of the data from the lowest 75%.											

The IHA direction, migration path length, and positions after bilateral facetectomy are displayed for the different preloads. Significant differences between the preload positions are marked with corresponding letters: c: central, d: dorsal, v: ventral, r: right, l: left. Deviations in the IHA directions in the sagittal and coronal planes were mostly observed for the ventral and lateral preloads (left, right). The IHA migration path length and positions did not differ between the preload positions.

### Instantaneous helical axis migration

Under intact conditions, the IHA under the central, dorsal, right, and left preloading conditions migrated from dorsal to ventral. The ventral preload position led to a movement from the left to the right, according to the axial rotation. The IHA migration path length was 4.4 (SD 2.9) mm for all the preload positions (Table 1, Figure 3).

After bilateral facetectomy, the IHA migration under the central preload condition was very low and on a punctual area. The ventral preload positioning showed a migration from the left to the right, according to the direction of the axial rotation. Lateral and dorsal preloads led to a migration from dorsal to ventral, and vice versa. On average, for all the preload positions, the IHA migration path length was 3.3 (SD 1.6) mm (Table 2, Figure 4). Bilateral facetectomy did not significantly change the IHA migration path compared to intact conditions (Figure 5C).

### Instantaneous helical axis direction

**Figure 6.**
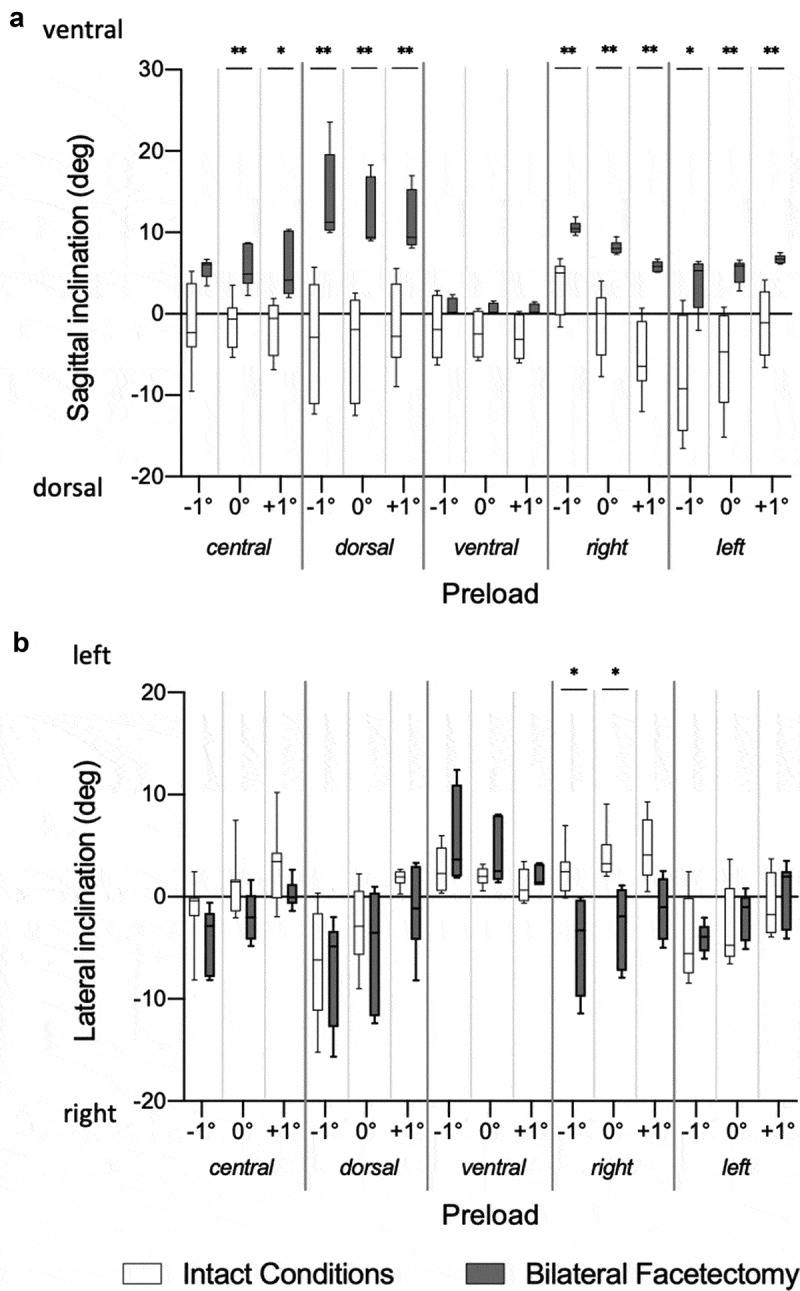
Differences in the IHA Direction. Differences in the HA direction in the sagittal (A) and coronal (B) planes between the intact conditions and after bilateral facetectomy are shown. The IHA direction in the sagittal plane is more ventrally inclined after bilateral facetectomy. Wilcoxon test, Dunn-Bonferroni correction for multiple testing, *: p < 0.05, **: p < 0.001

The direction of the IHA in the sagittal plane was nearly parallel to the coronal plane. A dorsal reclination was observed for axial rotations to the right and left side for certain preload conditions (Table 1, Figure 3). After bilateral facetectomy, the IHA direction in the sagittal plane showed a ventral inclination (Table 2, Figure 4). In comparison to the intact conditions, it was significantly more ventrally inclined under the dorsal, right, and left preload positionings during 0°, −1°, and +1° of axial rotation and under the central preload positioning at 0° and +1° (right) of axial rotation (Figure 6A).

**Figure 7.**
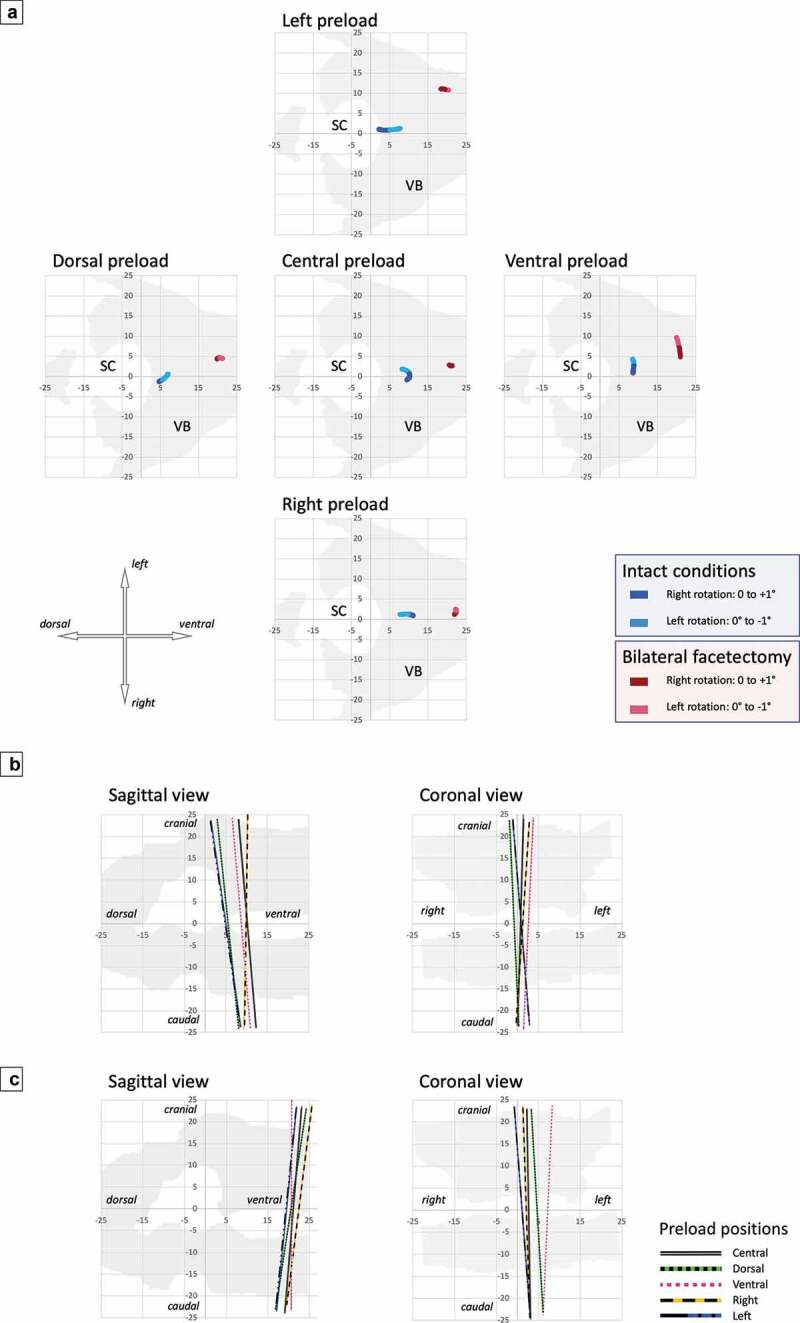
IHA Migration and Direction under Intact Conditions and after a Bilateral Facetectomy depicted in one of the four specimens as an example. **A** The IHA migration path of one of the four specimens is depicted under intact conditions (blue lines) and after a bilateral facetectomy (red lines) for all preload positions. Different color tones are used to define different directions of the axial rotation. **B** shows the IHA direction under intact conditions and **C** after bilateral facetectomy (0° axial rotation). Colors are used to define different preloads

The direction of the IHA in the coronal plane ran nearly parallelly to the sagittal plane under central preloading (Table 1, Figure 3). Under intact conditions an axial rotation to the right rather led to an IHA direction to the left, and vice versa. After bilateral facetectomy, an axial rotation to one side rather led to an IHA direction to the same side (Table 2, Figure 4). In comparison to the intact conditions, the IHA direction in the coronal plane was significantly more inclined rightwards, if a right preload and an axial rotation of 0° or −1° (left) was applied (Figure 6B).

### Individual analysis of one of the four specimens

The individual analysis of one of the four specimens reflects the key findings of the entire analysis (Figure 7). Under intact conditions, the IHA position was located at the posterior border of the vertebral body. After bilateral facetectomy, the IHA positions shifted ventrally by approximately 10 mm. While under intact conditions, the IHA migration path rather showed a movement from left to the right and vice versa under the central, dorsal and ventral preloads. Under the left or the right preload, the IHA rather migrated from dorsal to ventral and vice versa. Furthermore, after bilateral facetectomy, the IHA migration path was shorter compared to the distinctly longer IHA migration path under intact conditions, except for under the ventral preload where a longer IHA migration path length was observed for the intact conditions than after bilateral facetectomy. This might be a result of unloaded facet joints resulting in less guidance. In regard to the IHA direction in the sagittal plane, a dorsal reclination was observed under intact conditions. After bilateral facetectomy, this changed to a ventral inclination. The IHA direction in the coronal plane did not show a strong tendency to be inclined to one side. The entire analysis showed that, under intact conditions, an axial rotation to the right rather led to an IHA direction to the left and vice versa, while after bilateral facetectomy, an axial rotation to one side rather led to an IHA direction to the same side. This observation is only slightly indicated in the individual analysis.

## 4 Discussion

This study determined the direction and locations of the IHA in FSU T4-5 in axial rotation under intact conditions and after bilateral facetectomy. For the intact conditions, the results are in line with some recent studies that have observed the locations of the IHA in the anterior part of the spinal canal with a nearly vertical direction (Molnar et al. 2006; Liebsch and Wilke 2018). The bilateral facetectomy led to a ventral shift of the IHA locations and a ventral inclination of the IHA direction, indicating a substantial impact of FJs. The ‘absence’ of facet joints, physiologically contributing to the guidance of vertebral rotation, appears to show an interesting confirmation of a shift towards a purely discal mechanism of axial rotation after bilateral facetectomy. The results show that bilateral facetectomy leads to a ventral shift in the IHA locations of about 10 mm (intact conditions: 6.1 [SD 7.3] mm, after bilateral facetectomy: 18.7 [SD 4.0] mm). This considerable shift emphasizes the critical role of the FJs for the kinematics and FSU stability. The finding is strengthened by the dimensional size of the ventral shift in relation to the other results of the study. While the IHA direction and migration path length merely changed, the shift of the IHA locations was distinct (about 10 mm) after bilateral facetectomy.

Despite the recent results from Molnar et al. (2006) and Liebsch and Wilke (2018), who observed the locations of the IHA in the anterior portion of the spinal canal, a more holistic view including older references shows an inconsistency on IHA locations. IHA locations were observed at the dorsal end of the spinous process, the lateral end of the transverse process, and in between the anterior and posterior border of the vertebral body (Broc et al. 1997; Haher et al. 1992; Molnar et al. 2006). Molnar et al. (2006) combined three experimental designs (geometric calculation, analysis of changing volume of the spinal canal, and radiographic analysis) and concluded that the IHA locations are most likely in the anterior part of the spinal canal, as also noted by Liebsch and Wilke (2018). The results of the present study also determined the locations of the IHA very precisely in the anterior part of the spinal canal. Recently, Liebsch et al. (2020) analyzed the axis of rotation of eight thoracic FSUs of different segmental levels with an optical motion tracking system. In contrast to the previously assumed axis locations at the anterior part of the spinal canal (Molnar et al. 2006; Liebsch and Wilke 2018), in their study the average location was found in the vertebral body’s center (Liebsch et al. 2020). This axis location had been doubted because of the so-called ‘cigar cutting effect’ that would imply that the spinal cord was sheared off (Molnar et al. 2006; Liebsch and Wilke 2018). In light of the past contradictory results, the axis locations seem to be narrowed down and placed between the center of the vertebral body and the anterior part of the spinal canal.

In regard to preload positions the results show, that the dorsal preload position resulted in a more dorsal IHA location in comparison to the central preload position. It could be hypothesized that dorsal preloading creates more pressure on the FJs of the FSU, resulting in a continuous distinct contact of the joints shifting the IHA locations backwards (Wachowski et al. 2011). Conversely, under ventral preloading, the FJs no longer function properly due to less pressure on the surface of the joints. This unloading of FJ results in a rotation with less joint guidance and shifts the IHA location ventrally, that is line with the current literature of FJ biomechanics (Jaumard et al. 2011). Under the ventral preload position, the IHA migration formed a curve from one side to the other; under all other preloads, the IHA migrated on a curve from dorsal to ventral. This observation supports the thesis of a loss of FJ guidance under ventral preloading. Furthermore, the results showed an IHA location more toward the right under the dorsal preloading condition than under a ventral or right preloading condition. This asymmetrical effect might be the result of an FJ’s tropism (Jaumard et al. 2011). Pal et al. (2001) analyzed 30 specimens and could not observe statistical differences between the facet joint angles in the cervical and upper thoracic spine. Thus, with a larger study sample, this effect might have disappeared. Interestingly, Kuo et al. (2010) constructed a finite element lumbar spine model from a cadaveric specimen and analyzed the FJs’ contact behavior under different postures. They also observed a greater asymmetry of stress and strain distribution in the right FJs than in the left FJs.

In our study, the IHA ran nearly vertical with just a slight dynamic deviation within the sagittal and coronal planes; this finding is in line with the results reported by Liebsch et al. (2020). Under lateral preloading during intact conditions, the IHA inclination in the coronal plane was inclined in the opposite direction in relation to the applied preload (a right preload leads to a leftwards inclined IHA, and vice versa). Intriguingly, after bilateral facetectomy, the IHA inclination corresponds to the applied preload (the right preload leads to a rightwards inclined IHA). This strengthens the role of the FJs in guidance (Wachowski et al. 2009, 2017).

To conclude, the study determined precisely the location of the IHA under intact conditions at the anterior part of the spinal canal. The ventral shift of the IHA location and the alterations of the IHA direction after bilateral facetectomy show the substantial influence of the FJs on the kinematics of the FSU T4-5. Particularly, the distinct ventral shift of the IHA location after bilateral facetectomy indicates an impact of FJs and their capsules on the spinal sagittal stability. Regarding the clinical impact of this biomechanical study, the results have to be interpreted with caution. However, the observation supports the assumption that a sole bilateral facetectomy might result in spinal instability; therefore, combining a bilateral facetectomy with dorsal stabilization seems reasonable (Cusick et al. 1988; Zander et al. 2003).

This study observed the IHA characteristics in a very small range of rotation. It is noteworthy that each approach to analyzing the IHA has its own advantages and disadvantages. While the triad or the singular value decomposition seems better at describing the axis and the angle of rotation, the direct method seems more accurate at determining the intersection point of the helical axis with a plane of interest (Metzger et al. 2010). An optimized approach to gain more accuracy would be a hybrid method, i.e. a combination of the singular value decomposition and the direct approach as proposed by Metzger et al. (2010). In this study, a modified version of the direct approach to the determination of the IHA was used since the focus was the IHA migration in the horizontal plane (Mansour et al. 2004). Furthermore, as observed by Cescon et al. (2014), input noise can lead to error-prone calculations of position and direction of the IHA, in cases where very small IHA intervals are examined. The results of the present study base on IHA differences between very small intervals and might be affected by input noise. Thus, in this study, the exact values of IHA direction and orientation should be treated with caution to some extent. Nevertheless, the measurement of the differences between the intact conditions and the conditions after bilateral facetectomy are reliable since, under both conditions, the calculations were performed in the same manner.

This study has several limitations. First, a formalin-containing stabilizing solution was used. It has to be considered, that the use of formalin is supposed to have an impact on the stiffness of the intervertebral disc, ligaments, and FJ capsules (Wilke et al. 1996; Holewijn et al. 2017). Second, the specimens from younger donors might have altered the results. Facetectomies are performed in patients with neurological symptoms (O’Leary et al. 2018). Because sole nerve root impingements in the thoracic spine do not lead to motor deficits or disabilities resulting in dysfunction, the reason for such an operation is radicular pain irresponsive to conservative treatment (O’Leary et al. 2018). There is no specific statistical data on facetectomies in the thoracic spine. However, since age and comorbidities are risk factors for spinal surgery (Lee et al. 2012), the indication for a facetectomy in the thoracic spine seems less likely in older patients than in younger. Third, while the experiments were performed as quickly as possible, minimal damage to the specimen by the forces applied might have altered their kinematics. For better comparability, the same sequence of preload positions was used on each specimen and for both conditions. Nevertheless, the specimens might have had altered kinematics between the first and last preload positions. A randomization of preload positions in a larger sample could potentially solve this problem. Fourth, since the anterior and posterior longitudinal ligaments are long and attached semirigidly at each vertebra, the transection of these ligaments close to the studied FSU might alter the results if they are compared with the results of longer FSUs. Fifth, the removal of muscular tissue might have changed the results (Jaumard et al. 2011). Finally, using more specimens would have strengthened the results.

## Data Availability

Not applicable

## Exclusive Submission

The authors hereby declare that the manuscript, including related data, figures and tables has not been previously published and the manuscript is not under consideration elsewhere.

## References

[cit0001] Anderst W, Baillargeon E, Donaldson W, Lee J, Kang J. 2013. Motion path of the instant center of rotation in the cervical spine during in vivo dynamic flexion-extension: implications for artificial disc design and evaluation of motion quality after arthrodesis. Spine (Phila Pa 1976). 38(10):E594–601. doi:10.1097/BRS.0b013e31828ca5c7. 23429677 PMC3656913

[cit0002] Anderst WJ, West T, Donaldson WF 3rd, Lee JY, Kang JD. 2016. Longitudinal study of the six degrees of freedom cervical spine range of motion during dynamic flexion, extension, and rotation after single-level anterior arthrodesis. Spine (Phila Pa 1976). 41(22):E1319–E1327. doi:10.1097/BRS.0000000000001629. 27831986 PMC5119762

[cit0003] Broc GG, Crawford NR, Sonntag VK, Dickman CA. 1997. Biomechanical effects of transthoracic microdiscectomy. Spine (Phila Pa 1976). 22(6):605–612. doi:10.1097/00007632-199703150-00005. 9089932

[cit0004] Cescon C, Cattrysse E, Barbero M. 2014. Methodological analysis of finite helical axis behavior in cervical kinematics. J Electromyogr Kinesiol. 24(5):628–635. doi:10.1016/j.jelekin.2014.05.004. 24916306

[cit0005] Crawford NR, Baek S, Sawa AG, Safavi-Abbasi S, Sonntag VK, Duggal N. 2012. Biomechanics of a fixed-center of rotation cervical intervertebral disc prosthesis. Int J Spine Surg. 6(1):34–42. doi:10.1016/j.ijsp.2011.10.003. 25694869 PMC4300875

[cit0006] Cusick JF, Yoganandan N, Pintar F, Myklebust J, Hussain H. 1988. Biomechanics of cervical spine facetectomy and fixation techniques. Spine (Phila Pa 1976). 13(7):808–812. doi:10.1097/00007632-198807000-00017. 3194790

[cit0007] Dugailly PM, Sobczak S, Sholukha V, Van Sint Jan S, Salvia P, Feipel V, Rooze M. 2010. In vitro 3D-kinematics of the upper cervical spine: helical axis and simulation for axial rotation and flexion extension. Surg Radiol Anat. 32(2):141–151. doi:10.1007/s00276-009-0556-1. 19756350

[cit0008] Gautschi OP, Schatlo B, Schaller K, Tessitore E. 2011. Clinically relevant complications related to pedicle screw placement in thoracolumbar surgery and their management: a literature review of 35,630 pedicle screws. Neurosurg Focus. 31(4):E8. doi:10.3171/2011.7.FOCUS11168. 21961871

[cit0009] Haher TR, O’Brien M, Felmly WT, Welin D, Perrier G, Choueka J, Devlin V, Vassiliou A, Chow G. 1992. Instantaneous axis of rotation as a function of the three columns of the spine. Spine (Phila Pa 1976). 17(6 Suppl):S149–154. doi:10.1097/00007632-199206001-00015. 1631714

[cit0010] Holewijn RM, Faraj SSA, Kingma I, van Royen BJ, de Kleuver M, van der Veen AJ. 2017. Spinal biomechanical properties are significantly altered with a novel embalming method. J Biomech. 55:144–146. doi:10.1016/j.jbiomech.2017.02.012. 28259461

[cit0011] Jaumard NV, Welch WC, Winkelstein BA. 2011. Spinal facet joint biomechanics and mechanotransduction in normal, injury and degenerative conditions. J Biomech Eng. 133(7):071010. doi:10.1115/1.4004493. 21823749 PMC3705911

[cit0012] Kinzel GL, Hillberry BM, Hall AS Jr., Van Sickle DC, Harvey WM. 1972. Measurement of the total motion between two body segments. II. Description of application. J Biomech. 5(3):283–293. doi:10.1016/0021-9290(72)90045-0. 4666533

[cit0013] Kuo C-S, Hu H-T, Lin R-M, Huang K-Y, Lin P-C, Zhong Z-C, Hseih M-L. 2010. Biomechanical analysis of the lumbar spine on facet joint force and intradiscal pressure - a finite element study. BMC Musculoskelet Disord. 11(1):151. doi:10.1186/1471-2474-11-151. 20602783 PMC2913991

[cit0014] Lee MJ, Konodi MA, Cizik AM, Bransford RJ, Bellabarba C, Chapman JR. 2012. Risk factors for medical complication after spine surgery: a multivariate analysis of 1,591 patients. Spine J. 12(3):197–206. doi:10.1016/j.spinee.2011.11.008. 22245448 PMC3320089

[cit0015] Liebsch C, Jonas R, Wilke H-J. 2020. Thoracic spinal kinematics is affected by the grade of intervertebral disc degeneration, but not by the presence of the ribs: an in vitro study. Spine J. 20(3):488–498. doi:10.1016/j.spinee.2019.10.006. 31654810

[cit0016] Liebsch C, Wilke H-J. 2018. Chapter 3 - Basic Biomechanics of the Thoracic Spine and Rib Cage. In: Galbusera F, Wilke H-J, editors. Biomechanics of the Spine. Elsevier: Academic Press; p. 35–50. https://www.elsevier.com/books/biomechanics-of-the-spine/galbusera/978-0-12-812851-0?countrycode=DE&format=print&utm_source=google_ads&utm_medium=paid_search&utm_campaign=germanyshopping&gclid=Cj0KCQjwxdSHBhCdARIsAG6zhlVMiNJSDa8kTAbr5CfY3ztOgQpTPYXdjgrtCiTB2ZsvvNDHvLjdm20aAv9YEALw_wcB&gclsrc=aw.ds

[cit0017] Mansour M, Spiering S, Lee C, Dathe H, Kalscheuer AK, Kubein-Meesenburg D, Nagerl H. 2004. Evidence for IHA migration during axial rotation of a lumbar spine segment by using a novel high-resolution 6D kinematic tracking system. J Biomech. 37(4):583–592. doi:10.1016/j.jbiomech.2003.09.001. 14996572

[cit0018] Metzger MF, Faruk Senan NA, O’Reilly OM, Lotz JC. 2010. Minimizing errors associated with calculating the location of the helical axis for spinal motions. J Biomech. 43(14):2822–2829. doi:10.1016/j.jbiomech.2010.05.034. 20969997

[cit0019] Molnar S, Mano S, Kiss L, Csernatony Z. 2006. Ex vivo and in vitro determination of the axial rotational axis of the human thoracic spine. Spine (Phila Pa 1976). 31(26):E984–991. doi:10.1097/01.brs.0000250183.97746.51. 17172989

[cit0020] Nicholls FH, Bae J, Theologis AA, Eksi MS, Ames CP, Berven SH, Burch S, Tay BK, Deviren V. 2017. Factors Associated With the Development of and Revision for Proximal Junctional Kyphosis in 440 Consecutive Adult Spinal Deformity Patients. Spine (Phila Pa 1976). 42(22):1693–1698. doi:10.1097/BRS.0000000000002209. 28441308

[cit0021] O’Leary SA, Paschos NK, Link JM, Klineberg EO, Hu JC, Athanasiou KA. 2018. Facet Joints of the Spine: structure-Function Relationships, Problems and Treatments, and the Potential for Regeneration. Annu Rev Biomed Eng. 20:145–170. doi:10.1146/annurev-bioeng-062117-120924. 29494214

[cit0022] Oe S, Togawa D, Hasegawa T, Yamato Y, Yoshida G, Kobayashi S, Yasuda T, Banno T, Arima H, Mihara Y, et al. 2019. The Risk of Proximal Junctional Kyphosis Decreases in Patients With Optimal Thoracic Kyphosis. Spine Deform. 7(5):759–770. doi:10.1016/j.jspd.2018.12.007 31495477

[cit0023] Pal GP, Routal RV, Saggu SK. 2001. The orientation of the articular facets of the zygapophyseal joints at the cervical and upper thoracic region. J Anat. 198(Pt 4):431–441. doi:10.1046/j.1469-7580.2001.19840431.x. 11327205 PMC1468229

[cit0024] Park DK, Lin EL, Phillips FM. 2011. Index and adjacent level kinematics after cervical disc replacement and anterior fusion: in vivo quantitative radiographic analysis. Spine (Phila Pa 1976). 36(9):721–730. doi:10.1097/BRS.0b013e3181df10fc. 20543765

[cit0025] Roch PJ, Wagner M, Weiland J, Gezzi R, Spiering S, Lehmann W, Saul D, Weiser L, Viezens L, Wachowski MM. 2020a. Total disc arthroplasties change the kinematics of functional spinal units during lateral bending. Clin Biomech (Bristol, Avon). 73:130–139. doi:10.1016/j.clinbiomech.2020.01.007. 31982810

[cit0026] Roch PJ, Wagner M, Weiland J, Spiering S, Lehmann W, Saul D, Weiser L, Viezens L, Wachowski MM. 2020b. Total disc arthroplasties alter the characteristics of the instantaneous helical axis of the cervical functional spinal units C3/C4 and C5/C6 during flexion and extension in in vitro conditions. J Biomech. 100:109608. doi:10.1016/j.jbiomech.2020.109608. 31926589

[cit0027] Rousseau MA, Bradford DS, Hadi TM, Pedersen KL, Lotz JC. 2006. The instant axis of rotation influences facet forces at L5/S1 during flexion/extension and lateral bending. Eur Spine J. 15(3):299–307. doi:10.1007/s00586-005-0935-1. 16175392 PMC3489304

[cit0028] Rousseau MA, Cottin P, Levante S, Nogier A, Lazennec JY, Skalli W. 2008. In vivo kinematics of two types of ball-and-socket cervical disc replacements in the sagittal plane: cranial versus caudal geometric center. Spine (Phila Pa 1976). 33(1):E6–9. doi:10.1097/BRS.0b013e31815e5dce. 18165739

[cit0029] Schoenfeld AJ, Ochoa LM, Bader JO, Belmont PJ Jr. 2011. Risk factors for immediate postoperative complications and mortality following spine surgery: a study of 3475 patients from the National Surgical Quality Improvement Program. J Bone Joint Surg Am. 93(17):1577–1582. doi:10.2106/JBJS.J.01048. 21915571

[cit0030] Wachowski MM, Mansour M, Hawallek T, Kubein-Meesenburg D, Hubert J, Nägerl H. 2011. Parametric control of the stiffness of lumbar segments. Strain. 47(3):281–287. doi:10.1111/j.1475-1305.2009.00686.x.

[cit0031] Wachowski MM, Mansour M, Lee C, Ackenhausen A, Spiering S, Fanghanel J, Dumont C, Kubein-Meesenburg D, Nagerl H. 2009. How do spinal segments move? J Biomech. 42(14):2286–2293. doi:10.1016/j.jbiomech.2009.06.055. 19682692

[cit0032] Wachowski MM, Wagner M, Weiland J, Dorner J, Raab BW, Dathe H, Gezzi R, Kubein-Meesenburg D, Nagerl H. 2013. Does total disc arthroplasty in C3/C4-segments change the kinematic features of axial rotation? J Biomech. 46(10):1739–1745. doi:10.1016/j.jbiomech.2013.03.027. 23659912

[cit0033] Wachowski MM, Weiland J, Wagner M, Gezzi R, Kubein-Meesenburg D, Nagerl H. 2017. Kinematics of cervical segments C5/C6 in axial rotation before and after total disc arthroplasty. Eur Spine J. 26(9):2425–2433. doi:10.1007/s00586-017-5073-z. 28378073

[cit0034] Wilke HJ, Krischak S, Claes LE. 1996. Formalin fixation strongly influences biomechanical properties of the spine. J Biomech. 29(12):1629–1631. doi:10.1016/S0021-9290(96)80016-9. 8945663

[cit0035] Zander T, Rohlmann A, Klockner C, Bergmann G. 2003. Influence of graded facetectomy and laminectomy on spinal biomechanics. Eur Spine J. 12(4):427–434. doi:10.1007/s00586-003-0540-0. 12720068 PMC3467787

